# Quantifying the major mechanisms of recent gene duplications in the human and mouse genomes: a novel strategy to estimate gene duplication rates

**DOI:** 10.1186/gb-2007-8-8-r158

**Published:** 2007-08-02

**Authors:** Deng Pan, Liqing Zhang

**Affiliations:** 1 Department of Computer Science, Virginia Tech, Torgerson Hall, Blacksburg, Virginia 24061-0106, USA

## Abstract

**Background:**

The rate of gene duplication is an important parameter in the study of evolution, but the influence of gene conversion and technical problems have confounded previous attempts to provide a satisfying estimate. We propose a new strategy to estimate the rate that involves separate quantification of the rates of two different mechanisms of gene duplication and subsequent combination of the two rates, based on their respective contributions to the overall gene duplication rate.

**Results:**

Previous estimates of gene duplication rates are based on small gene families. Therefore, to assess the applicability of this to families of all sizes, we looked at both two-copy gene families and the entire genome. We studied unequal crossover and retrotransposition, and found that these mechanisms of gene duplication are largely independent and account for a substantial amount of duplicated genes. Unequal crossover contributed more to duplications in the entire genome than retrotransposition did, but this contribution was significantly less in two-copy gene families, and duplicated genes arising from this mechanism are more likely to be retained. Combining rates of duplication using the two mechanisms, we estimated the overall rates to be from approximately 0.515 to 1.49 × 10^-3 ^per gene per million years in human, and from approximately 1.23 to 4.23 × 10^-3 ^in mouse. The rates estimated from two-copy gene families are always lower than those from the entire genome, and so it is not appropriate to use small families to estimate the rate for the entire genome.

**Conclusion:**

We present a novel strategy for estimating gene duplication rates. Our results show that different mechanisms contribute differently to the evolution of small and large gene families.

## Abstract 2

By studying two mechanisms of gene duplication, unequal crossover and retrotranspostion, and looking at both small gene families and the entire genome, a new estimate for the rate of gene duplication is made which is more accurate for both small and large gene families.

## Background

Gene duplication is among the major mechanisms providing raw materials that give rise to new genes and functions [1,2]. The duplication of genes is thought to be a continual process in evolution. However, despite numerous studies of gene duplication, the fundamental issue of how frequently gene duplication occurs is still unresolved.

To estimate the gene duplication rate, one must first determine how to distinguish young duplicated genes from old ones. To solve this problem, two methods were proposed in previous studies. The first method is to use K_s _(the synonymous distance) [3] or other neutral markers [4] as the time proxy to define newly born duplicates. This method was first used by Lynch and Conery [3] to estimate gene duplication rates in the genomes of yeast, *Drosophila*, and *Caenorhabtidis elegans*. However, the neutrality of K_s _was questioned by later studies [4-7]. Accordingly, Gu and coworkers [4] proposed that a combination of K_s _and other neutral markers, such as intron and flanking regions, should be used to estimate gene duplication rates. However, although the marker is neutral and the molecular clock model holds, the first method still has problems. One of these is that it cannot distinguish true newly born duplicates from old duplicates that appear to be young because of gene conversion. Gene conversion is a homogenizing process between two homologous DNA fragments that occurs during recombination by transferring DNA sequence information from one fragment to another. Thus, the divergence between two DNA fragments can decrease dramatically following gene conversion. Because gene conversion occurs frequently in the genome [8,9], this first method can yield inflated estimates of rate.

To overcome this problem, Gao and Innan [10] proposed a phylogeny-based method that does not rely on the molecular clock model. This second method effectively eliminates erroneous detection of old duplicates as young ones and reduces the influence of gene conversion. Consequently, the duplication rate in yeast estimated by Gao and Innan [10] is much lower than that by Lynch and Conery [3]. However, the phylogeny-based method is not perfect either. One of its limitations is that it is computationally difficult when it is applied to large gene families, and it becomes even more so when gene loss is taken into account. This is probably why Gao and Innan [10] only studied two-copy gene families, which represent a small fraction of duplicated genes in the yeast genome. In fact, Lynch and Conery [3] also limited their study to just the families with fewer than five members in order to minimize the influence of gene conversion. Can duplication rates estimated from small gene families represent the rate for the entire genome?

Here, we propose a new strategy to estimate the rate of gene duplication. A major obstacle to the estimation is difficulty in minimizing the effect of gene conversion while taking large families into account. Both methods used in previous studies consider gene duplication as a single entity, ignoring the fact that gene duplication is actually achieved by multiple mechanisms. Major mechanisms of gene duplication are unequal crossover, retroposition, and genome duplication (including large segmental duplication) [11]. It is known that genes generated by different duplication mechanisms have different sensitivities to gene conversion. For instance, tandem duplications (generated by unequal crossover) in large gene families are believed to have been extensively affected by gene conversion [8], whereas those generated by retroposition are not. This inspired us to estimate the total duplication rate by considering the duplication rates achieved by the different mechanisms. The new strategy has at least two advantages over previous methods. First, we can estimate rates of gene duplication for duplicated genes that are not sensitive to gene conversion by using the neutral time proxies (such as K_s_) directly, even for large gene families. Second, for the duplicated genes that are highly sensitive to gene conversion, we can take into account the specific features of the genes and make adjustments to achieve better control over the influence of gene conversion.

To implement our new strategy, we must know the relative contributions made by each mechanism to gene duplication. Unfortunately, despite numerous studies on gene duplication, almost all of the available studies focus on one mechanism of duplication at a time. It is interesting that almost all of these studies concluded that the focal mechanism is the dominant one. Among the three well known major mechanisms of gene duplication, genome duplication was first emphasized by Ohno [1], who claimed that it is the main process of gene duplication in vertebrates. His hypothesis finds supports from the 2R hypothesis in vertebrates, which posits that there might have been two rounds of genome duplication in vertebrates [12-14]. However, this hypothesis was challenged by several recent genome-wide studies [15-18], in which a large proportion of gene duplications in the human and mouse genomes was found to be tandemly aligned and unequal crossover appeared to be the driving force. Indeed, our previous study [19] also indicated that tandemly arrayed genes (TAGs) account for about 20% of all genes in mammals. Because TAGs are among the primary products of unequal crossover [20], it appears likely that unequal crossover is a dominant mechanism of gene duplication. On the other hand, retroposition is also thought to play an important role in gene duplication [21,22]. Retroposition is an RNA-mediated process that occurs through reverse transcribing the mRNA of a gene and inserting the resulting cDNA into the genome. Once a retrocopy recruits regulatory elements by chance after insertion and acquires a new function, it becomes a retrogene. A significant number of retrogenes have been reported in many organisms [23-29]. It is evident that we must consider various duplication mechanisms at the same time if we are to understand their relative contributions to duplications in the genome.

As a first step, we quantified the respective contributions made by unequal crossover and retroposition to recently duplicated genes. We focused on these two mechanisms because for the following four reasons. First, no matter whether the 2R hypothesis holds, the last possible genome duplication in vertebrates occurred more than 400 million years (MY) ago [30], and so its contribution to recent gene duplications is negligible. Second, recent segmental duplications cover only about 2% of the mouse genome [31] and 4% of the human genome [32], and usually do not contain genes [33]. Third, small segmental duplications can also be generated by unequal crossover. Fourth, within some large segmental duplication regions, there exist micro-duplications that are generated by unequal crossover or retroposition caused by the more frequent occurrence of unequal crossover and retroposition than large segmental duplication. Also, the genes generated by these micro-duplication events cannot be regarded as contributions of large segmental duplication. Therefore, the contribution of large segmental duplication to recent gene duplications is expected to be small, and therefore we focus on the two remaining major mechanisms of gene duplication.

In this study, we compared the relative contributions made by unequal crossover and retroposition to duplications in the human and mouse genomes, and estimated the respective duplication rates of the two mechanisms. We conducted our analysis in both two-copy gene families and in the entire genome in order to test whether the rates estimated from two-copy families can represent that for the entire genome. We hope that the results of this study will further our understanding of the mechanisms of gene duplication in mammals.

## Results

In order to examine whether gene duplication rates estimated from small gene families can be used to represent duplication rates in the entire genome, we estimated rates using two sets of data: all duplicated genes in the entire genome (denoted as the ALL gene set) and only the duplicated genes in the two-copy gene families (denoted as the FAM2 gene set). Therefore, the FAM2 gene set is a subset of the ALL gene set (Additional data files 1 to 4 provide lists of genes in ALL and FAM2).

We used K_s _as a proxy to time the duplication events. K_s _has been criticized for not being strictly neutral in yeast, *Drosophila*, and *C. elegans*, among other organisms [4]. This should not be a critical problem in the present study for the following reasons. First, comparison of human and chimp orthologous genes indicates that although more than 90% of the synonymous mutations are under very weak selection, most of them are too weak to influence the substitution rate [34]. Second, the effective population size of mammals is believed to be much smaller than those of nonmammalian species. Therefore, with small selective coefficients (s) and small population sizes (N), most of the synonymous mutations are expected to be effectively neutral (2Ns << 1). Wyckoff and coworkers [35] showed that even for the very conserved ribosomal protein genes, the K_s _between human and mouse is essentially identical to the average K_s _of the entire human-mouse orthologous gene set.

### Relative contributions of unequal crossover and retroposition to gene duplication

**Figure 1:**
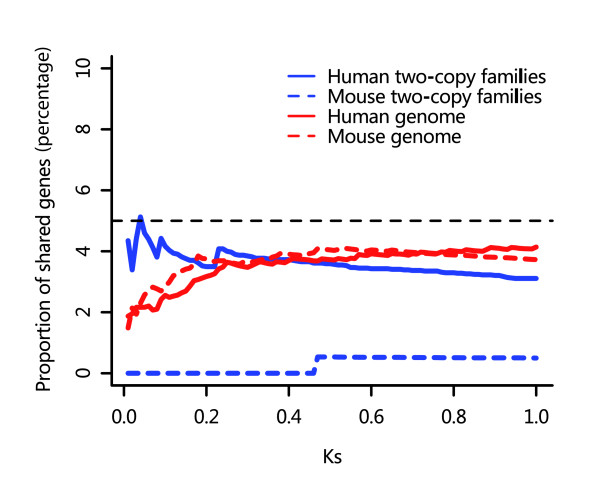
Duplicated genes belong to both TAGs and retrogenes. The proportion of shared genes is the proportion of duplicated genes that belong to both tandemly arrayed genes (TAGs) and retroposed genes as a function of K_s_.

Theoretically, unequal crossover and retroposition are two independent biologic processes, but this has not been tested empirically in genome-wide studies. To address this issue, we plotted the distribution of the percentage of genes that belong to both TAGs and retroposed genes as a function of K_s _(Figure 1). For both species, even when the least stringent criteria are used for TAG and retrogene identification, the percentages in all K_s _bins are still no more than about 5% in both two-copy families and the entire genome, indicating that the two processes are indeed independent.

**Figure 2:**
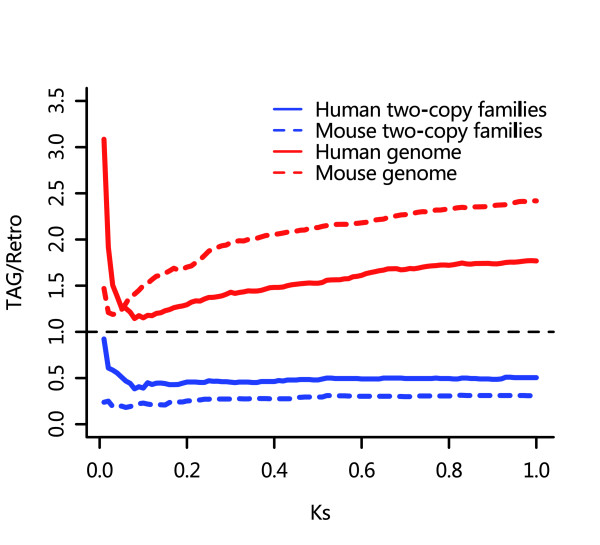
Relative contribution of unequal crossover and retroposition. 'TAG/Retro' is the ratio of the cumulative number of tandemly arrayed genes (TAGs) to retroposed genes as a function of K_s_.

Because duplication by unequal crossover and that by retroposition are largely independent of each other, we can compare the relative contributions made by these two mechanisms to gene duplication by simply calculating the ratio of TAGs to retroposition-related genes. The distribution of the ratio of TAGs to retroposition-related genes as a function of K_s _(Figure 2) shows that, generally, the ratios in two-copy gene families (always <1) are much lower than those in the entire genome (always >1) in both species, suggesting that unequal crossover is more active in large gene families but less active in small ones than retroposition. Figure 2 is based on the stringent TAG definition and the lower limit of retrogene numbers. Other criteria yield similar patterns. In a recent study (unpublished data), we found that retroposition is not directly correlated with the size of gene family. Interestingly, in all cases, the ratios are very high initially and decrease sharply as K_s _increases from 0 to about 0.05 to 0.1. This could be caused by either an excess of young TAGs caused by gene conversion or by a lack of retrogenes in small K_s _bins.

### Gene duplications via unequal crossover

**Figure 3:**
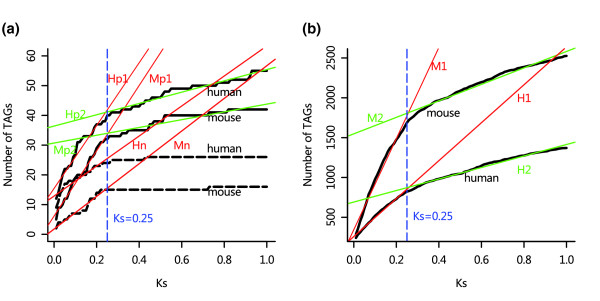
Gene duplication rate via unequal crossover. The rates are the slopes of the linear functions (colored lines) fitted to the curves of the cumulative distributions of tandemly arrayed genes (TAGs). Parameter estimates of the linear functions are shown in Table 1. **(a) **TAGs in two-copy families. The NEW gene set is plotted in bold broken lines, the linear functions of which are H_n _and M_n _(red). The FAM2 gene set was plotted in bold lines, the linear functions of which are H_p1 _and M_p1 _(red) for the part with K_s _≤ 0.25, and H_p2 _and M_p2 _(green) for the part with K_s _> 0.25. **(b) **TAGs in the entire genome. The linear functions are H_1 _and M_1 _(red) for the part with K_s _≤ 0.25, and H_2 _and M_2 _(green) for the part with K_s _> 0.25.

We plotted the cumulative distributions of the number of TAGs as a function of K_s _(Figures 3a,b). We divided the curves into two parts using K_s _= 0.25 as the cut-off and fitted linear models to each part of the curves. The results are shown in Table 1. The slopes of the linear functions are therefore the estimates of gene duplication rates for the two types of duplication mechanisms. In both species, rates of TAG duplication decrease at K_s _≥ 0.25 for both the FAM2 gene set and the ALL gene set. According to Lynch and Cornery [3], gene loss should have occurred extensively before K_s _= 0.25. However, the distributions appear to imply that gene loss in TAGs does not occur soon after duplication events, which means newly generated TAGs are more likely to be preserved for a long time.

**Table 1:** Parameter estimates for the linear functions (y = mx + b) in Figures 3 and 4

Species	Mechanisms	Gene set	Functions	Parameters			
				
				m	b	*r*^a^	*P *value
Human	TAG	NEW	H_n_	50.46	12.88	0.94	7.0 × e^-12^
		FAM2	H_p1_	105.62	15.43	0.97	4.0 × e^-15^
			H_p2_	19.00	36.41	0.98	<2.2 × e^-16^
		ALL	H_1_	2,381.20	258.50	0.99	<2.2 × e^-16^
			H_2_	722.30	691.50	0.99	<2.2 × e^-16^
	Retro	FAM2	H_pr_	730.00	6.90	0.99	9.5 × e^-04^
		ALL	H_r_	2,840.00	18.00	0.99	4.9 × e^-04^
Mouse	TAG	NEW	M_n_	54.93	1.78	0.98	<2.2 × e^-16^
		FAM2	M_p1_	109.77	6.13	0.99	<2.2 × e^-16^
			M_p2_	13.09	30.71	0.93	<2.2 × e^-16^
		ALL	M_1_	5,717.80	343.10	0.99	<2.2 × e^-16^
			M_2_	1,034.00	1548.00	0.99	<2.2 × e^-16^
	Retro	FAM2	M_pr_	1,000.00	14.60	0.98	2.9 × e^-03^
		ALL	M_r_	3,750.00	71.30	0.99	1.1 × e^-03^

^a^Pearson correlation coefficient. TAG, tandemly arrayed gene.

Because it has been shown that TAGs are highly affected by gene conversion, to explore the region where the true duplication rate in TAGs will be located, we determined recently duplicated genes in two-copy families using a phylogeny-based method similar to that used by Gao and Innan [10] (the collection of these genes is denoted as the NEW gene set; see Materials and methods, below, for detail and Additional data files 5 and 6 for the gene list). Thus, genes in the NEW gene set should truly be recently born in the human or mouse lineage, rather than results of gene conversion on older duplicates. About 94% of the human gene pairs and 91% of the mouse gene pairs in the NEW gene set have K_s _≤ 0.25, which confirms the recent duplications of these genes. The majority of the gene pairs in the NEW gene set have K_a_/K_s _< 1, which suggests that these genes are mostly under purifying selection (see Additional data file 7). The cumulative distributions of TAGs in the NEW gene set are plotted in Figure 3a. Because most of the genes in the NEW gene set have K_s _≤ 0.25, we only used these genes for curve fitting. It shows that the slopes of the linear functions of the NEW gene set (H_n _and M_n_) are located between the slopes of the two parts of the FAM2 gene set (H_p1 _and H_p2 _in human; M_p1 _and M_p2 _in mouse), which means that in two-copy gene families the real TAG duplication rate is located between the slopes of the two parts of the curves.

Theoretically, we can perform a similar analysis for the ALL gene set. In practice, however, it is extremely difficult to identify recently duplicated genes in large gene families using the phylogeny-based method. However, we noticed that the patterns of distributions of TAGs with respect to K_s _are very similar between the two-copy families and the entire genome, and in particular the K_s _divergence points for rate changes are both around 0.25. Therefore, we believe that, for the entire genome, the real TAG duplication rate is also located between the slopes of the two parts of the curves. This is based on the following reasoning. Let R_t _be the true gene duplication rate, R_oi _the observed gene duplication rate, R_ci _the gene conversion rate, and R_li _the gene loss rate, where *I *= 1 when K_s _≤ 0.25 and i = 2 when 0.25 < K_s _≤ 1. Then, R_oi _= R_t _+ R_ci _- R_li_. For the first part of the curves, as shown above, the rates of gene loss in TAGs should be low, especially immediately after the duplication events [3], but gene conversion in TAGs is supposedly strong [8,9] and always in effect. So, we have R_c1 _> R_l1_, and then R_o1 _> R_t_. For the second part of the curves, gene conversion is greatly weakened because of high sequence divergence; meanwhile, the net effect of gene loss is greater than the first part of the curves, especially because of the fact that many TAGs can become superficially lost (fail to be classified as TAGs) as a result of various genome rearrangements [18]. So we have R_c2 _< R_l2 _and then R_o2 _< R_t_. Thus, R_o1 _> R_t _> R_o2_. Also, because TAGs make a greater contribution to gene duplication in large families than in small ones (Figure 2), gene conversion should be more active in large gene families than in small ones. It is therefore likely that R_t _for the entire genome is closer to R_o2 _than it is in two-copy gene families.

We converted the slopes of the linear functions to obtain absolute rates. For the two-copy gene families, we used the slopes for the NEW gene sets directly, whereas for the entire genome we used the two slopes of the linear functions for the ALL gene sets as the lower and upper estimates of the rates. Assuming a synonymous substitution rate of 1 to 1.3 × 10^-9 ^per site per year for human [36] and 2 to 2.6 × 10^-9 ^per site per year for mouse [37], and 8,312 and 8,105 singleton genes in the human and mouse genomes, respectively, we estimated the rates of gene duplication in two-copy gene families to be 0.012 to 0.016 × 10^-3 ^per gene per MY in human and 0.027 to 0.035 × 10^-3 ^per gene per MY in mouse. For the entire genome, assuming the same substitution rates, and 19,032 in human and 20,453 in mouse to be the effective numbers of genes before one duplication event per genome (see Materials and methods, below), we estimated rates of duplication for the entire genome to be 0.076 to 0.325 × 10^-3 ^per gene per MY in human and 0.202 to 1.45 × 10^-3 ^per gene per MY in mouse. Therefore, the rates estimated for the entire genome are approximately 5 to 27 times faster than the rates estimated for two-copy gene families in human, and 6 to 54 times faster in mouse.

The above rates are all based on the stringent TAG definition, which allows only up to one spacer gene in the array. If the nonstringent TAG definition is used, then for the two-copy gene families the rates are about 0.015 to 0.020 × 10^-3 ^per gene per MY in human and 0.041 to 0.053 × 10^-3 ^in mouse; for the entire genome, the rates are 0.083 to 0.406 × 10^-3 ^per gene per MY in human and 0.217 to 1.71 × 10^-3 ^in mouse. The rates are similar to those obtained under the stringent TAG definition, showing that the results are not very sensitive to the number of spacers allowed.

### Gene duplications via retroposition

**Figure 4:**
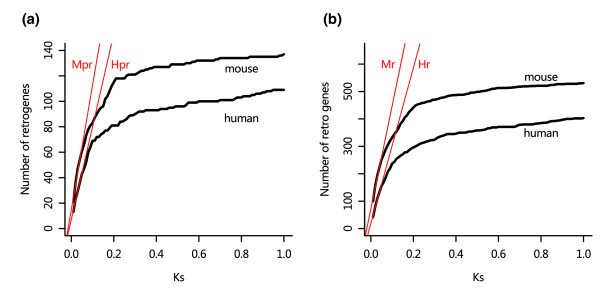
Gene duplication rate via retroposition. The rates are the slopes of the linear functions (red lines) fitted to the curves of the cumulative distributions of retrogenes. All of the linear functions are fitted to the part of the curves with K_s _≤ 0.05. Parameters of the linear functions are shown in Table 1. **(a) **Retrogenes in two-copy families. The linear functions are H_pr _and M_pr_. **(b) **Retrogenes in the entire genome. The linear functions are H_r _and M_r_.

Retrogenes were screened for the two genomes. Because of uncertainty regarding the number of multi-retroposition events in large gene families, we determined upper and lower limits for the number of retrogenes (see Materials and methods, below, for details). There are 585 putative parental-retrogene pairs in human and 727 in mouse if one takes all of the possible multi-retroposition events as one event for each parental gene, or 700 putative parental-retrogene pairs in human and 857 in mouse if one includes all of those possible multi-retroposition events. The actual number of retrogenes should be within these ranges. The cumulative distributions of the numbers of retrogenes as a function of K_s _are shown in Figures 4a,b.

Ezawa and coworkers [9] demonstrated that most of the gene pairs that underwent gene conversion are linked on the same chromosomes in mouse. Because most of the retrogenes in our data are located on different chromosomes from their parental genes (Table 2), we believe that gene conversion has little influence on retrogenes. Thus, unlike the case for TAGs, we simply used the retrogenes in the small K_s _regions (K_s _≤ 0.05) to estimate the rate of gene duplication for retroposition. According to Lynch and Cornery [3], there should be no apparent gene loss within K_s _= 0.05.

**Table 2:** Chromosomal locations of parental-retrogene pairs

Species	Types	NEW	FAM2	ALL^a^
Human	Intra-chromosomal	8	14	116
		(29.6%)	(9.9%)	(19.8%)
	Inter-chromosomal	19	128	469
		(71.4%)	(90.1%)	(80.2%)

Mouse	Intra-chromosomal	6	21	151
		(11.8%)	(12%)	(20.8%)
	Inter-chromosomal	45	154	576
		(88.2%)	(88%)	(79.2%)

Percentages are given in parentheses. ^a^Based on the lower limit of the number of retropositions; the upper limit provides similar results.

Using the same rate transformation procedures as for TAGs, we estimated the retrogene formation rate to be 0.176 to 0.228 × 10^-3 ^per gene per MY in human and 0.393 to 0.642 × 10^-3 ^per gene per MY in mouse for the two-copy gene families, and 0.298 to 0.388 × 10^-3 ^per gene per MY in human and 0.733 to 0.953 × 10^-3 ^per gene per MY in mouse for the entire genome. The rates estimated for two-copy gene families are still about 1.3 to 2.2 times lower than those for the entire genome in human and 1.1 to 1.9 times lower in mouse, but the contrast between the rates for two-copy families and the rates for the entire genome is much smaller than that of TAGs, which is consistent with the observation that the retrogene formation is more active in two-copy gene families than larger families (Figure 2).

### Recent gene duplication rates

Because unequal crossover and retroposition are independent, we can sum the two rates from these two mechanisms. Assuming mechanisms other than these two are also independent, we can derive the overall gene duplication rates using the following equation:

$$\text{R}=\frac{{\text{R}}_{\text{u}}+{\text{R}}_{\text{r}}}{\text{W}}$$

Where R_u _and R_r _are gene duplication rates caused by unequal crossover and retroposition, respectively; and W is the total percentage of the duplicated genes involved in these two processes. Because R_u _and R_r _are estimated using different K_s _regions, the intersecting K_s _regions should be used to estimate R. Because the influence of gene conversion is greatly reduced when K_s _> 0.25, we used K_s _= 0.25 as the point at which to estimate W and the range of K_s _< 0.25 for estimating R_u _and R_r _(Table 3). In fact, there is little change in W for 0.25 ≤ K_s _≤ 1.

**Table 3:** Summary of duplication rates

Categories	Human		Mouse	
	
	Two-copy gene families	Entire genome	Two-copy gene families	Entire genome
TAG rate (R_u_)	0.012 to 0.020	0.076 to 0.406	0.027 to 0.053	0.202 to 1.71
Retro rate (R_r_)	0.176 to 0.228	0.298 to 0.388	0.494 to 0.642	0.733 to 0.953
Total weight (W)	61.0% to 73.2%	53.3% to 72.6%	68.5% to 77.2%	62.9% to 76.3%
R_u _+ R_r_	0.188 to 0.248	0.374 to 0.794	0.521 to 0.695	0.935 to 2.66
Gene duplication Rate (R)	0.257 to 0.407	0.515 to 1.49	0.675 to 1.01	1.23 to 4.23

The rates are expressed as × 10^-3 ^per gene per million years. The lower and upper limits are calculated through all combinations of different tandemly arrayed gene (TAG) or retrogene identification criteria

All of the gene duplication rates estimated thus far are summarized in Table 3. Recent tandem duplication rates are more than ten times slower than retrogene formation rates for two-copy families, but the contrast in rates of duplication for these two mechanisms becomes less obvious for the entire genome. The rates estimated using two-copy gene families are about 1.2 to 6 times lower than those using the whole genome in both species. The duplication rates in mouse are much higher than those in human.

## Discussion

Gene duplication has been studied extensively. However, most studies focus on one duplication mechanism at a time or take all of the duplication mechanisms as a whole and do not consider the differences between the various mechanisms. In this study we considered the relative extent to which the various mechanisms contribute to recent gene duplications in human and mouse, and we estimated the gene duplication rate occurring via different duplication mechanisms. To achieve our goals, we studied unequal crossover and retroposition simultaneously. We quantitatively confirmed that these two processes are independent and compared their respective contributions to gene duplications. These results provide the basis of our novel strategy for estimating gene duplication rates.

In our new strategy, gene duplication rates are estimated separately for unequal crossover and retroposition, and later the two rates are combined to estimate the overall gene duplication rate. Because gene conversion has minimal effect on the divergence of retrogenes, we are confident that the estimates of rates of duplication by retroposition are reliable. In fact, using the rates of duplication by retroposition alone to estimate the overall rates of gene duplication also gives an estimate that is of the same magnitude as the combined rate estimates from the two duplication mechanisms. Also, by taking advantage of the fact that frequencies of gene conversion reduce with the divergence of TAGs, we were able to control the influence of gene conversion to a predictable range, even for large gene families. Therefore, our new method appears promising. However, there are still several issues that must be addressed. First, as stated above, there might be some problems with K_s _as a time proxy in organisms with large population size. We should therefore use other, more neutral markers in the organisms with large population size if possible. Second, our screening method for retrogenes has limited power to identify chimeric retrogenes, and it is therefore likely that rates of duplication by retroposition are underestimated in our study. Third, one may argue that, according to our strategy, a similar estimate of overall rate could be achieved by considering just one mechanism, combined with knowledge of its relative contribution; however, the more mechanisms used, the more robust will be the rate achieved.

We used the total weight W (the percentage of duplicated genes that are either TAGs or retrogenes) to transform the sum of R_u _and R_r _into the overall gene duplication rate R for the genome. As shown in Table 3, even with the most stringent criteria in the identification of TAGs and retrogenes, W is more than 53%. On average, W is about 60% to 70% in human and mouse, suggesting that unequal crossover and retroposition are the major mechanisms for generating gene duplications. The remaining duplicated genes may be generated by recent large segmental duplications, nonallelic homologous recombination [38], and even mechanisms that are yet to be identified. It is also possible that some of the duplicated genes generated by unequal crossover and retroposition were not detected by our screening method. Genes generated by unequal crossover can be rearranged to different chromosomes as a result of genome rearrangement, and our method will not be able to identify them. Also, retrogenes can gain new introns and exons and become multiple exon genes, and our method will not be able to identify them either. It should also be mentioned that our way of combining the rate components through W is very simple and may be biased if W is not correctly estimated. More sophisticated ways to combine the components in the final rate should be studied in the future.

Our final rate estimation of *R *is about 0.515 to 1.49 × 10^-3 ^per gene per MY in human and about 1.23 to 4.23 × 10^-3 ^in mouse (Table 3). These rates are in the range of the estimates reported by Lynch and Conery [3] (2 to 20 × 10^-3 ^per gene per MY), in which families with no more than five members were used for estimation in fly, yeast, and worm. However, Gao and Innan [10] proposed an estimate of the gene duplication rate in yeast that is two orders of magnitude lower than that estimated by Lynch and Conery [3]. Because Gao and Innan used a phylogeny-based method to obtain the data, they claimed that the lower rates are due to the removal of the effect of gene conversion on the data. However, our results show that most of the statistics in two-copy gene families exhibit different behaviors from those in the whole genome, and gene duplication rates estimated in two-copy gene families are generally lower than those estimated from the entire genome, even after taking gene conversion into account. Therefore, the much lower rate proposed by Gao and Innan [10] may in part be due to the usage of two-copy families. However, because the species used in their study and ours are different, more work should be done to test this hypothesis.

The comparison of different mechanisms enables us to gain more insight into the relative importance of different mechanisms of gene duplication and dynamics of duplicated genes generated by these different mechanisms. Our results show that genes generated by unequal crossover are more likely to be preserved than retrotransposed copies. The K_s _cut-off for the slowdown of the observed duplicated gene formation rates in TAG (about 0.25) is much larger than that of retrogenes (about 0.05). This phenomenon is largely because of the influence of gene conversion.

Apart from duplication rates, we also compared the absolute numbers of genes involved in unequal crossover and retroposition with respect to K_s _divergence of duplicated genes. The results show that unequal crossover generally contributes more than retroposition to gene duplications in the entire genome, and the difference will be larger as divergence becomes larger (Figure 2). The longer half-life of TAGs appears to ensure that more TAGs will be preserved in the genome. However, the situation in two-copy families is different. Retroposition-related genes generally occur more than twice as frequently as TAGs in human, and more than three times as frequently as in mouse. The excess of retroposition-related genes in two-copy families indicates that retroposition plays a major role in generating two-copy gene families from singleton genes. It also means that singleton genes are less likely to change into a TAG of two-members, which may be because unequal crossover is less likely to occur in a single copy gene than in an existing TAG because of the lack of sequence similarity. Note that small gene families can also come from large gene families as a result of gene loss. Here, we only consider the overall net effect.

The genomes of rodents change faster than those of primates [31,39-41]. Accordingly, we also found that the gene duplication rates, either via unequal crossover or via retroposition, are higher in mouse than in human, which probably reflects the intrinsic difference between the two species. A recent study [37] proposed a more important role of positive selection than for the duplication-degeneration-complementation (DDC) model [42] in maintaining more gene duplications in mouse than in human. However, the DDC model cannot be used to explain duplications by retroposition. The higher preservation rate of retrogenes in mouse may still be due to adaptive evolution, because mouse has a much larger effective population size than human, which means natural selection in mouse is generally stronger than that in human. However, this hypothesis requires testing in the future.

## Materials and methods

### Data compiling

We retrieved all data from Ensembl (version 41) using BioMart. Altogether, there are 31,206 and 27,964 genes in the human and mouse genomes, respectively. We focused on the genes that are nuclear protein coding and for which the chromosome location is known. We used the longest transcripts of those genes having multiple spliced forms. We discarded genes encoding proteins shorter than 50 amino acids to ensure annotation quality and obtained 22,598 human genes and 24,064 mouse genes. Of these, 8,312 in human and 8,105 in mouse are single-copy genes, and the remaining are clustered by Ensembl into 3,538 families in human and 3,600 families in mouse.

We paired genes within each family and aligned the DNA sequences of these gene pairs based on the corresponding protein alignments using ClustalW [43]. We required the overlapping percentage of the alignment in each gene pair to be no less than 70%, and we obtained 88,423 gene pairs (containing 12,782 genes) in human and 127,146 gene pairs (containing 14,382 genes) in mouse. This is our entire dataset, which represents all duplicated genes in the two genomes denoted as the ALL gene set for clarity. Furthermore, we retrieved genes from the ALL gene set that are in two-copy gene families, denoted as the FAM2 gene set. There are 1,364 and 1,323 gene pairs in human and mouse, respectively, in the FAM2 gene set.

In order to evaluate the influence of gene conversion in two-copy families, we compiled a gene set (denoted NEW) from the FAM2 gene set using a phylogeny-based method without assuming the molecular clock model. We chose outgroup species as reference points to identify recently duplicated genes. We used five sequenced mammalian genomes: dog (*Canis familiaris*), cattle (*Bos Taurus*), rat (*Rattus norvegicus*), macaca (*Macaca mulatta*), and opossum (*Monodelphis domestica*) as outgroups. (Also, human or mouse was used as an outgroup, depending on which species was the focal species.) We identified the gene pairs in human (or mouse) that have at most one gene in the outgroup species belonging to the same gene family (Ensembl families were defined based on sequence similarity). There are 118 human gene pairs and 120 mouse gene pairs that satisfy this criterion. We then manually examined each gene pair using the Ensembl GeneTreeView Browser to confirm the phylogeny and discarded genes that are most likely false positives of recent duplications. Finally, we obtained 108 newly born duplicated gene pairs in human and 108 pairs in mouse.

We computed K_a _(the number of nonsynonymous substitutions per nonsynonymous site) and K_s _(the number of synonymous substitutions per synonymous site) for all gene pairs by a maximum likelihood method using PAML [44,45] and performed subsequent analysis on all three datasets.

### Screening TAGs

TAGs are tandemly arrayed genes that belong to the same gene family. There are sometimes spacers within a TAG, which are genes that do not belong to the same family as the TAG members. Similar to work by Shoja and Zhang [19], we used two TAG definitions: the stringent TAG definition with 0 ≤ S ≤ 1 and the nonstringent definition with 0 ≤ S ≤ 10, where S is the number of spacer genes. Specifically, we sorted genes by their chromosomes and indexed them in ascending order based on their physical locations. Let d denote the absolute difference in the indices between two genes on the same chromosome. If d ≤ 2, then two genes belong to a TAG according to the stringent definition; if d ≤ 11, then two genes belong to a TAG according to the nonstringent definition. We then clustered two-gene TAGs into larger TAGs by using a single linkage cluster algorithm. We screened TAGs for each dataset under each TAG definition in each of the species.

The distributions of the cumulative number of duplicated genes in TAGs as a function of K_s _were plotted in R [46] in both two-copy gene families and in the entire genome. The interval of the data points in terms of K_s _of the curves is 0.01. Because initially genes are singletons and the duplication direction in TAGs is unknown, the number of duplicated genes were calculated as the total number of genes in TAGs in each case minus the number of initial singleton genes, which can be estimated as one half of the number of genes in two-copy gene families.

### Screening retrogenes

We retrieved gene structure information from Ensembl and merged introns shorter than 40 nucleotides [26]. We considered gene pairs with a multiple exon member (the parental gene) and an intronless member (the derived retrogene) as putative parental-retrogene pairs. Because intron loss or gain seldom occurs in mammals [47], it is unlikely that the putative retrogenes are due to intron loss and the parental genes are due to intron gain. We ignored those pairs that have intronless parental genes. However, this is a minor problem because, for instance, in two-copy gene families there are only seven gene pairs (about 3.4%) with K_s _≤ 0.25 in which both members are intronless and located on different chromosomes (most of the retropositions occur inter-chromosomally; Table 2). Our screening method for retrogenes has limited power to identify chimeric retrogenes, but that will not affect our results very much because we are only interested in the number of gene duplication events.

Because of multiple mappings between putative parental genes and retrogenes in large families, we picked out parental-retrogene pairs using the following procedures. First, because a retrogene has only one parental gene, when an intronless gene is paired with several multi-exon genes, we selected the pair that has the smallest K_s _as the target pair and obtained 700 pairs in human and 857 pairs in mouse. Of these, there still exist gene pairs whose parental genes are mapped to multiple retrogenes. Because the likelihood of intron gain is low [47], these pairs can be the result of either multiple retropositions (scenario 1), one retroposition followed by multiple duplications of the retrogene (scenario 2), or a mixture of these two scenarios. It is therefore very difficult to determine precisely the number of retrogene formation events. To be as broad as possible, we considered both upper and lower limits: 700 in human and 857 in mouse (corresponding to scenario 1), and 585 in human and 727 in mouse (corresponding to scenario 2). We obtained the lower limits by keeping only the pair that has the smallest K_s _among all of the gene pairs that share the same parental genes. The number of retrogenes in human in this study is approximately the same as that reported by Marques and coworkers [26]. Similarly, we also plotted the distribution of cumulative number of retrogenes as a function of K_s _using R [46]. The interval of the data points in terms of K_s _of the curves is 0.01.

### Estimating rates

Cumulative distributions of the numbers of duplicated genes generated by unequal crossover and retroposition were plotted as a function of K_s_. Gene duplication rates were estimated by curve fitting to a linear model. The slopes of the linear models are essentially the estimates of observed gene duplication rates per genome per synonymous substitution, and the intercepts are estimates of the numbers of duplicated genes observed per genome when K_s _approaches 0. All of the curve fitting and statistical tests were performed in R [46]. The curves of TAGs are separated into two parts using K_s _= 0.25 as a cutoff and linearly fitted separately. The K_s _cut-off at 0.25 is based on the distributions in Figure 3a,b. Unlike the case of TAGs, we only used one line to fit retrogene curves with K_s _≤ 0.05 because the influence of gene conversion on retrogenes is minimal.

To convert duplication rates per genome to duplication rates per gene, we must know the effective number of genes (N_g_) before one duplication event per genome. For two-copy gene families, N_g _is the number of singletons (8,312 in human and 8,105 in mouse). For families of all sizes, N_g _is calculated as the total number of genes per genome minus the number of gene families, which are 19,032 in human and 20,453 in mouse.

### Other analyses

All of the text parsing and processing procedures were performed using a series of programs written in the OCAML language [48]. Data were loaded into a MySQL database for subsequent querying.

## Additional data files

The following additional data are available with the online version of this paper. Additional data file 1 provides the human ALL gene set. Additional data file 2 provides the mouse ALL gene set. Additional data file 3 provides the human FAM2 gene set. Additional data file 4 provides the mouse FAM2 gene set. Additional data file 5 provides the human NEW gene set. Additional data file 6 provides the mouse NEW gene set. Additional data file 7 provides the distribution of K_a_/K_s _to K_s _of the gene pairs in the NEW gene set.

## Authors' contributions

DP designed, analyzed and wrote the paper. LZ designed and wrote the paper.

## Supplementary Material

Additional data file 1 Provided is the human ALL gene set. Click here for file

Additional data file 2 Provided is the mouse ALL gene set. Click here for file

Additional data file 3 Provided is the human FAM2 gene set. Click here for file

Additional data file 4 Provided is the mouse FAM2 gene set. Click here for file

Additional data file 5 Provided is the human NEW gene set. Click here for file

Additional data file 6 Provided is the mouse NEW gene set. Click here for file

Additional data file 7 Provided is the distribution of *K*_*a*_/*K*_*s *_to *K*_*s *_of the gene pairs in the NEW gene set. Click here for file
